# Effects of Bushen Tianjing Recipe in a rat model of tripterygium glycoside-induced premature ovarian failure

**DOI:** 10.1186/s13020-017-0131-3

**Published:** 2017-04-24

**Authors:** Xiaofeng Xu, Yong Tan, Guorong Jiang, Xuanyi Chen, Rensheng Lai, Lurong Zhang, Guoqiang Liang

**Affiliations:** 1 Department of Gynecology, Suzhou Hospital Affiliated to Nanjing University of Chinese Medicine, No. 18 Yangsu Road, Gusu District, Suzhou, 215009 Jiangsu Province China; 2 Department of Gynecology, The No.1 Clinical Medical College, Nanjing University of Chinese Medicine, No. 138 Xianlin Avenue, Xianlin University City, Nanjing, 210046 Jiangsu Province China; 3 Institute of Traditional Chinese Medicine, Suzhou Hospital Affiliated to Nanjing University of Chinese Medicine, No. 18 Yangsu Road, Gusu District, Suzhou, 215009 Jiangsu Province China; 4 Department of Pathology, Jiangsu Hospital of Traditional Chinese Medicine, No.155 Hanzhong Road, Qinhuai District, Nanjing, 210002 Jiangsu Province China

**Keywords:** Bushen Tianjing Recipe, Premature ovarian failure, Tripterygium glycoside, Angiogenesis, Apoptosis

## Abstract

**Background:**

Bushen Tianjing Recipe (BTR) is a traditional Chinese herbal medicine that has been prescribed for premature ovarian failure (POF) for decades in China. Nevertheless, little is known regarding its underlying molecular mechanism. In the present study, we investigated the effects of BTR in a tripterygium glycoside (TG)-induced-POF rat model.

**Methods:**

Three doses of BTR were administered via intragastric gavage to adult female Sprague–Dawley (SD) rats with TG-induced POF. After 15 days of treatment, the estrous cycle was examined by vaginal smear analysis. Serum levels of estradiol, follicle-stimulating hormone, progesterone, and testosterone were measured by radioimmunoassay. Histological analysis and assessment of apoptosis were performed after hematoxylin and eosin staining of ovarian tissue sections. The expression of vascular endothelial growth factor (VEGF), vascular endothelial growth factor receptor 2 (VEGFR2), anti-apoptotic factor Bcl-2, and pro-apoptotic factors Bax and caspase 3 in ovaries of animals was examined by an immunohistochemistry process.

**Results:**

BTR not only reverted an abnormal estrous cycle and decreased the ovary index in POF rats but also improved the abnormal secretion of reproductive hormones associated with POF. In addition, treatment with BTR can protect ovaries from TG-induced damage, induce intraovarian expression of VEGF and VEGFR2, and regulate intraovarian expression of apoptosis-related proteins.

**Conclusions:**

Our results show that BTR is effective in the treatment of TG-induced POF rats. Promotion of angiogenesis and anti-apoptosis are most likely to contribute to the effects of BTR against POF.

**Electronic supplementary material:**

The online version of this article (doi:10.1186/s13020-017-0131-3) contains supplementary material, which is available to authorized users.

## Background

Primary hypogonadism is defined as ovarian insufficiency accompanied by a high serum level of follicle stimulating hormone (FSH). In women, one of the most common forms of primary hypogonadism is premature ovarian failure (POF), which is also known as premature menopause [1]. POF occurs in 1% of all women and in 0.1% of women younger than 30 years [2]. It may be caused by either an increased rate of follicle loss, a decreased number of follicles being formed during ovarian development, or follicles unresponsive to hormonal stimulation [3]. The loss of fertility and the clinical effects of hypoestrogenism are the two significant consequences of POF [4], which are manifested by amenorrhea, elevated gonadotropin levels, and an irregular menstrual cycle [5]. Previous studies have regarded POF as a disease with a heterogenous pathogenic background, and the known etiologic factors of POF include genetic abnormalities, autoimmune disease, and environmental insults, as well as iatrogenic impairment following surgery, radiotherapy, and pharmacotherapy [6, 7].

Management of POF essentially involves hormone replacement therapy (HRT), and infertility treatment. HRT can compensate for the estrogen deficiency in POF patients, consequently relieving their menopausal symptoms [8, 9]. However, concerns have been raised regarding this therapy due to the increased risk of breast cancer, heart attack, and stroke [7]. Therefore, HRT is usually a last resort for POF and the lowest dose for the shortest period of time should be employed [10]. Infertility is a significant issue for most patients suffering from POF. Recently, a number of treatment regimens, including clomiphene, gonadotropins, gonadotropin-releasing hormone (GnRH) agonists, and immunosuppressants, have been used clinically with the aim of restoring fertility [11, 12]. However, these treatment approaches have limited success in improving the likelihood of conception as well as ameliorating menopausal symptoms [13]. Thus, there is still an increasing demand for novel and effective therapeutics for POF.

In recent years, Traditional Chinese Medicine (TCM) has attracted significant attention for the management of female reproductive dysfunctions due to its efficacy, safety, and low cost [14–18]. Bushen Tianjing Recipe (BTR) is a traditional Chinese herbal medicine that has been prescribed for female reproductive disorders including POF for decades in China. Recently, several clinical studies demonstrated the clinical efficacy of BTR. For example, Liang et al. [19] reported that in patients undergoing in vitro fertilization-embryo transfer (IVF-ET), administration of BTR could enhance the quality of oocytes, and increase the sensitivity of ovarian follicles to exogenous gonadotropins. In another randomized clinical trial, the combination of BTR and HRT showed significant therapeutic effects in patients with POF, which was manifested by substantial improvements in clinical symptoms, menstrual states, and serum sex hormones levels [20]. Taken together, these findings provide a rationale for implicating the therapeutic use of BTR in the treatment of POF and other female reproductive disorders. Nevertheless, little is known regarding the underlying molecular mechanism.

BTR contains four components, *Rehmanniae Radix Praeparata*, *Paeoniae Radix Alba*, *Testudinis Carapax et Plastrum,* and *Corni Fructus*. In vitro and in vivo studies have revealed that the major bioactive compounds isolated from these components have ovarian failure-resistant effects or pharmacological activities against gynaecological disorders [21, 22]. More importantly, pro-angiogenic and anti-apoptotic signaling cascades have been implicated in the bioactivities of these compounds [23–26]. Therefore, we hypothesized that the effect of BTR against POF may be mediated through pro-angiogenic and anti-apoptotic mechanisms. To validate this hypothesis, in the present study, we investigated the effects of BTR as well as the underling mechanisms in a tripterygium glycoside (TG)-induced POF rat model.

## Methods

The Minimum Standards of Reporting Checklist (Additional file 1) contains details of the experimental design, and statistics, and resources used in this study.

### Chemicals and reagents

BTR was provided by Suzhou Chunhui Traditional Chinese Herbal Medicine Factory (Suzhou, China). The components of BTR and their amounts are listed in Table 1. The preparation process of BTR was as described in the following.

**Table 1:** The components of Bushen Tianjing Recipe

Components	Chinese name	Origin	Amount used (g)
Rehmanniae Radix Praeparata	Shu Di Huang	*Rehmannia glutinosa*	10
Testudinis Carapax et Plastrum	Gui Jia	*Chinemys reevesii*	10
Paeoniae Radix Alba	Bai Shao	*Paeonia lactiflora*	10
Corni Fructus	Shan Yu Rou	*Cornus officinalis*	10

All components were mixed in proportion and then decocted with an eightfold quantity of water (volume/weight) twice, for 1.5 h each time. The resultant decoction was filtrated, combined, and concentrated to a relative density of 1.2 g/ml (at 60 °C) under vacuum. The concentrated decoction (i.e., BTR) was stored at 4 °C for future use. According to the Pharmacopoeia of the People’s Republic of China (2010 edition) and the Quality Specifications of Chinese Traditional Medicine of Jiangsu Province, high performance liquid chromatogram (HPLC) and thin-layer chromatography (TLC) methods were employed to control the quality of BTR (Table 2; Additional file 2: Figures S1–S4, Table S1).

**Table 2:** Quality evaluation of BTR

Major constituents	Method of determination	Quality specifications
Catalpol	HPLC	>50 mg per 10 g BTR
Paeoniflorin	HPLC	>0.2 g per 10 g BTR
Morroniside	HPLC	>50 mg per 10 g BTR
*Testudinis Carapax et Plastrum*	TLC	Arginine contained

*HPLC* high performance liquid chromatogram, *TLC* thin-layer chromatography

TG tablets (10 mg) were purchased from Hunan Qianjin Xieli Pharmaceutics Co., Ltd. (Batch No.: 20090102, Zhuzhou, China) and dissolved in sterile distilled water to yield a 50 mg/ml solution. Immunoradioassay kits for rat estradiol (E2) (Catalog No.: B05JFB), progesterone (P) (Catalog No.: B08JFB), FSH (Catalog No.: B03TFB), and testosterone (T) (Catalog No.: B10TLB) were obtained from Beijing Beifang Medical & Bioengineering Co., Ltd. (Beijing, China). Immunohistochemistry (IHC) kits for detection of vascular endothelial growth factor (VEGF) (Catalog No.: LYM00952) and vascular endothelial growth factor receptor-2 (VEGFR2) (Catalog No.: LYM00761) were obtained from Lanzhou Yijian Medical Co., Ltd. (Lanzhou China). IHC kits for Bax (Catalog No.: BA0315-1), Bcl-2 (Catalog No.: BA0412), and caspase-3 (active form, Catalog No.: BA2142) were provided by Wuhan Boster Bio-engineering Co., Ltd. (Wuhan, China).

### Animals and POF induction

The protocol of the animal study was approved by the Animal Ethics Committee of Nanjing University of Traditional Chinese Medicine (Nanjing, China) (Approval No. JSSZUSPF/SQ-11). All animal care and treatment were conducted in strict accordance with institutional guidelines. Adult female Sprague–Dawley (SD) rats (weighing 180–220 g, aged 12 weeks) were purchased from Shanghai Slac Laboratory Animal Technology Co., Ltd. (Shanghai, China) and housed in an air-conditioned facility with a room temperature of 25 ± 1 °C, humidity of 50 ± 5%, and a 12-h light/dark cycle. All animals were supplied with food and water ad libitum. Prior to experiments, they were allowed to acclimate to the animal facility for 1 week. Then, vaginal smear analysis was performed every day for 10 days to screen 50 animals with normal estrous cycle (defined as 4–6 days in length) as experimental subjects [27]. The screened animals were randomly divided into the following five groups of ten animals each: the control group, the POF model group, the BTR-low group, the BTR-medium group, and the BTR-high group. Drug dosage and administration schedules for each group of animals are summarized in Table 3. The doses of BTR selected for animals were based on human equivalent doses calculated by the method of Reagan-Shaw et al. [28].

**Table 3:** Drug dose and administration schedule for each group of animals

Group	Drug dose and administration schedule
Control	4 ml 0.9% saline twice daily (9:00 a.m. and 3:00 p.m.), i.g., for 15 days
POF model	TG at 50 mg/kg, i.g. at 9:00 a.m. plus 4 ml 0.9% saline, i.g. at 3:00 p.m., for 15 days
BTR-low	TG at 50 mg/kg, i.g. at 9:00 a.m. plus BTR at 1.88 g/kg, i.g. at 3:00 p.m., for 15 days
BTR-medium	TG at 50 mg/kg, i.g. at 9:00 a.m. plus BTR at 3.75 g/kg, i.g. at 3:00 p.m., for 15 days
BTR-high	TG at 50 mg/kg, i.g. at 9:00 a.m. plus BTR at 7.50 g/kg, i.g. at 3:00 p.m., for 15 days

*POF* premature ovarian failure, *TG* tripterygium glycoside, *BTR* Bushen Tianjing Recipe, *i.g.* intragastric gavage, *AM* in the morning, *PM* in the afternoon

### Assessment of estrous cycles and sample collection

During the administration period, the estrous cycle of each animal was examined by vaginal smear analysis according to a previously described method [29]. The length of a cycle was determined as the number of days between two non-consecutive days during which estrus cytology was observed [30]. The length of estrous cycle ≥15 days was defined as cessation of cycle. For each animal, the mean length of estrous cycle was calculated when the number of estrous cycles was ≥2. After 15 days of administration, animals with estrous cycles (based on vaginal smear analysis) were sacrificed at the proestrus stage (within 1–5 days after the last administration); while those with cessation of cycle were sacrificed on the day after the last administration. Before sacrifice, blood samples were collected from the femoral artery of the animals. After sacrifice, both ovaries were surgically removed and weighed by an electronic balance. The ovary index for each animal was calculated according to the following formula: ovary index = the wet weight of bilateral ovaries (mg)/body weight (g) × 100%.

### Measurement of serum levels of E2, FSH, P, and T

For each animal, the serum levels of E2, FSH, P, and T were measured by γ-radioimmunoassay using commercialized immunoradioassay kits (Beijing Beifang Medical & Bioengineering Co., Ltd.) in accordance with the manufacturer’s instructions. Each assay was performed in duplicate and the mean vale was calculated.

### Histological analysis

For histochemical analysis, the left ovary of each animal was fixed in 4% neutral buffered paraformaldehyde, embedded in paraffin, and sliced into 3–5 μm sections. The sections were then stained with hematoxylin and eosin (H&E) according to standard protocols. Slides were viewed and photographed under 100× magnification using an Olympus BX-51 microscope (Olympus, Tokyo, Japan). For quantitative assessment of apoptosis, five sections were randomly selected for each rat and photographed at 400×. Subsequently, a total of ten non-overlapping high power fields (HPFs) were randomly chosen from these sections for counting the number of apoptotic granulosa cells. The counting was conducted by two independent analysts who were blinded to the treatment assignment, and the average values were recorded. Then, the average value across all HPFs was calculated for each animal.

### Immunohistochemical analysis

IHC analysis was conducted to detect expression levels of VEGF, VEGFR2, Bcl-2, Bax, and caspases 3 in ovarian sections from different treatment groups. Briefly, the right ovary of each animal was fixed in 4% neutral buffered paraformaldehyde solution for 2 h. Then, the fixed samples were dehydrated in gradient alcohol solutions, embedded in paraffin, and cut into 3–5 μm sections. Then, IHC staining was performed using commercialized IHC kits according to the manufacturers’ instructions. Finally, the sections were visualized with 3,3′-diaminobenzidine (DAB), counterstained using hematoxylin, and photographed at 100×. For an individual rat, the immunoreactivity of VEGF, VEGFR2, Bcl-2, Bax, and caspases 3 was analyzed in ten random HPFs from five sections and scored using a semi-quantitative scoring system for staining intensity as previously described [31]: 0, no staining; 1, weak staining; 2, moderate staining; and 3, strong staining. Scoring was performed by an experienced pathologist who was blinded to the treatment assignment using an automatic IHC image analysis software (https://imagej.nih.gov/ij/plugins/ihc-toolbox/index.html). Then, the median value across all HPFs was calculated for each animal.

### Statistical analysis

Statistical analysis was conducted using SPSS 13.0 software (SPSS Inc., Chicago, IL, USA). All continuous data were tested for normal distribution using the D’Agostino-Pearson omnibus test. Data with normal distribution are presented as mean ± standard deviation (SD). Data with skewed distribution are presented as median and interquartile range (IQR, range from the 25th to the 75th percentile). Statistical differences between means were assessed using one-way analysis of variance (ANOVA), followed by Tukey’s post hoc multiple comparison tests. Statistical differences between medians were assessed with the nonparametric Kruskal–Wallis test followed by Dunn’s post hoc multiple comparison tests. *P* < 0.05 was considered statistically significant.

## Results

### BTR reverted abnormal estrous cycle and improved the ovary index in a TG-induced POF rat model

**Fig. 1:**
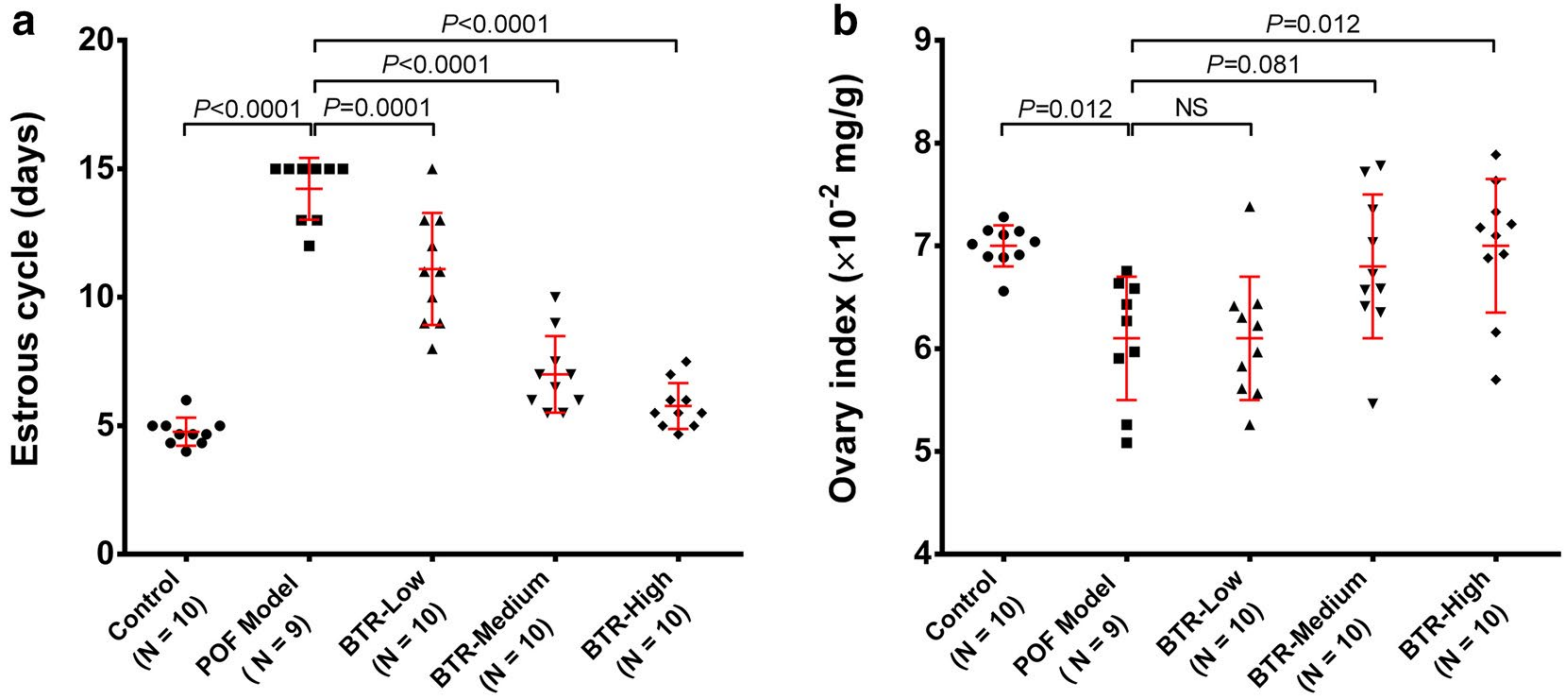
The length of the estrous cycle (**a**) and ovary index (**b**) in different treatment groups. Each *dot* in **a** and **b** represents the length of estrous cycle during the 15-day administration period and the ovary index at the end of administration for an individual animal, respectively. When the number of estrous cycles ≥2, the mean value is presented. *Bars* and *error bars* are means and SD. All statistical analyses were performed using ANOVA followed by Tukey’s post hoc test. *NS* not significant

During the experiment, one animal in the POF model group died of unknown causes, and its data were discarded from subsequent analyses. First, we examined the estrous cycle and ovary index of the animals from different groups. After 5 days of TG induction, the POF model group showed a decrease in food intake, locomotor activity, and stimulus response, compared to the control group. For the three BTR-treated groups, however, the food intake of the animals remained unchanged, and no abnormal behavior was observed during the entire course of the experiment. Within 15 days of TG induction, the majority of the animals (6 of 9) in the POF model group displayed cessation of the estrous cycle and the rest showed a significantly longer estrous cycle duration than the control animals (*P* < 0.0001) (Fig. 1a). However, this TG-induced abnormality in the estrous cycle was significantly counteracted by BTR in a dose-dependent manner. Specifically, the animals treated with a high dose of BTR displayed similar estrous cycle duration as the control group.

By the end of treatment, anatomical examination of the ovaries was performed and the ovary index was calculated for each animal in all groups. As shown in Fig. 1b, the ovary index in the POF model group was significantly decreased, compared to the control group (*P* = 0.012). However, this TG-induced decrease in the ovary index could be prevented by different concentrations of BTR in a dose-dependent manner. Taken together, these findings indicate that BTR can revert abnormal estrous cycle and improve the ovary index in a TG-induced POF rat model.

### Effect of BTR on serum levels of E2, FSH, P, and T in a TG-induced POF model

Under the POF condition, the ovary fails to function normally in response to appropriate gonadotropin stimulation, and thus doesn’t produce normal amounts of sex hormones [7]. Previous studies have reported that POF is associated with a decreased serum level of E2 and increased serum levels of P, FSH, and T [32]. We therefore measured serum levels of E2, FSH, P, and T in the animals from different groups to investigate the effect of BTR on secretion of these reproductive hormones. As shown in Table 4, animals in the POF model group showed a dramatically lower serum level of E2 and considerably higher levels of FSH, P, and T compared with the control group. However, these TG-induced changes were significantly prevented by BTR in a dose-dependent manner, suggesting that BTR can improve the abnormal secretion of reproductive hormones associated with POF.

**Table 4:** Effect of Bushen Tianjing Recipe on serum levels of E2, FSH, P, and T in a TG-induced rat POF model

Group	Number of animals	E2 (pg/ml)^a,b^	FSH (μg/ml)^a,b^	P (ng/ml)^a,b^	T (pg/dl)^a,b^
Control	10	643.9 ± 157.9	2.52 ± 0.59	0.65 ± 0.52	6.97 ± 2.52
POF model	9	355.8 ± 130.6*	8.56 ± 2.13**	0.95 ± 0.21*	21.68 ± 9.61**
BTR-low	10	364.1 ± 79.8	7.85 ± 1.62	0.82 ± 0.18^#^	12.17 ± 3.20^##^
BTR-medium	10	553.1 ± 107.5^##^	3.52 ± 0.75^##^	0.59 ± 0.56^#^	10.60 ± 4.33^##^
BTR-high	10	578.6 ± 129.0^##^	3.57 ± 0.70^##^	0.52 ± 0.15^#^	7.71 ± 1.24^##^

*E2* estradiol, *FSH* follicle-stimulating hormone, *P* progesterone, *T* testosterone, *POF* premature ovarian failure, *BTR* Bushen Tianjing Recipe

^a^Data are presented as mean ± SD

^b^* *P* < 0.05 and ** *P* < 0.01 versus the control group; ^#^ *P* < 0.05 and ^##^ *P* < 0.01 versus the POF model group

### Effect of BTR on primary and maturing follicles as well as luteal function in TG-induced POF model

**Fig. 2:**
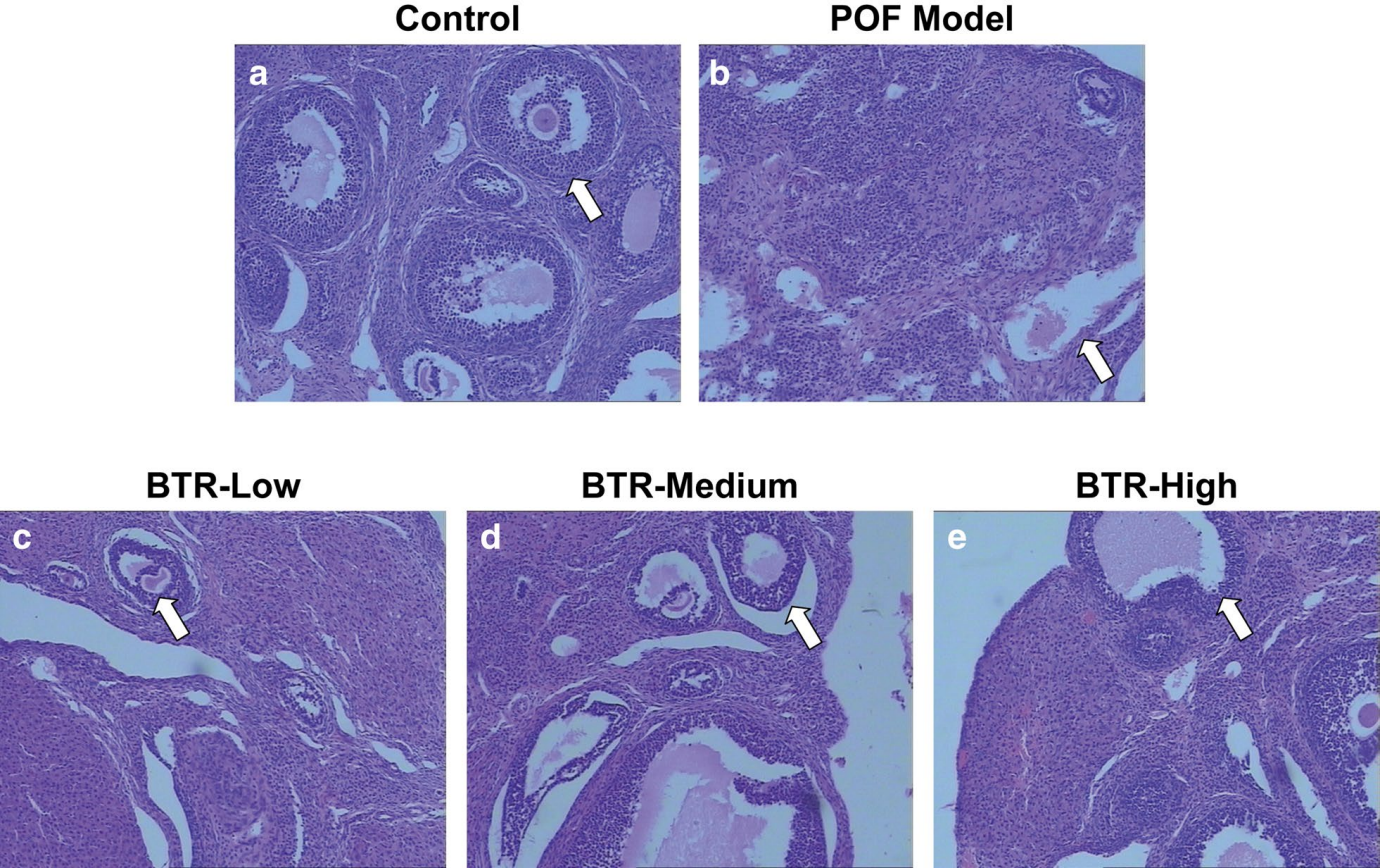
Representative hematoxylin and eosin-stained images of histological sections from all experimental groups (**a**–**e**). A decrease in the number of primary follicles (as indicated by *white arrows*) can be seen in the POF model group, while treatment with Bushen Tianjing Recipe achieved a significant improvement in the follicular count. Magnification ×100

POF is manifested by a decrease in the number of developing follicles and consequently affects reproductive activity [33]. Therefore, we performed histological analyses of ovarian sections to investigate the effect of BTR on primary and growing follicles and luteal function in TG-induced POF model. The H&E stained sections of the control group showed the normal histologic structure of the cortex and medulla with multiple maturing follicles at different stages (Fig. 2a). The corpus luteum could be seen, but neither follicular ovarian cysts nor corpus luteum hematomas were observed. Additionally, no infiltration of inflammatory cells or ovarian fibrosis was found in either the cortex or medulla. In the sections from the POF model group, however, an abnormal histology of the cortex and medulla was observed with a markedly reduced number of primordial and primary follicles, and few developing and mature follicles with degenerated oocytes (Fig. 2b; Additional file 2: Figure S5). In addition, ovarian interstitial fibrosis, as well as regression and necrosis of the corpus luteum accompanied by inflammatory cell infiltration and vessel dilation could be distinguished in the sections. For the three BTR-treated groups, the sections showed that the TG-induced histopathological changes were significantly alleviated by treatment with BTR (Fig. 2c–e). Specifically, the BTR-medium and -high groups showed almost normal histology comparable to that of the control group.

### Effect of BTR on intraovarian expression of VEGF and VEGFR2 in a TG-induced POF rat model

**Fig. 3:**
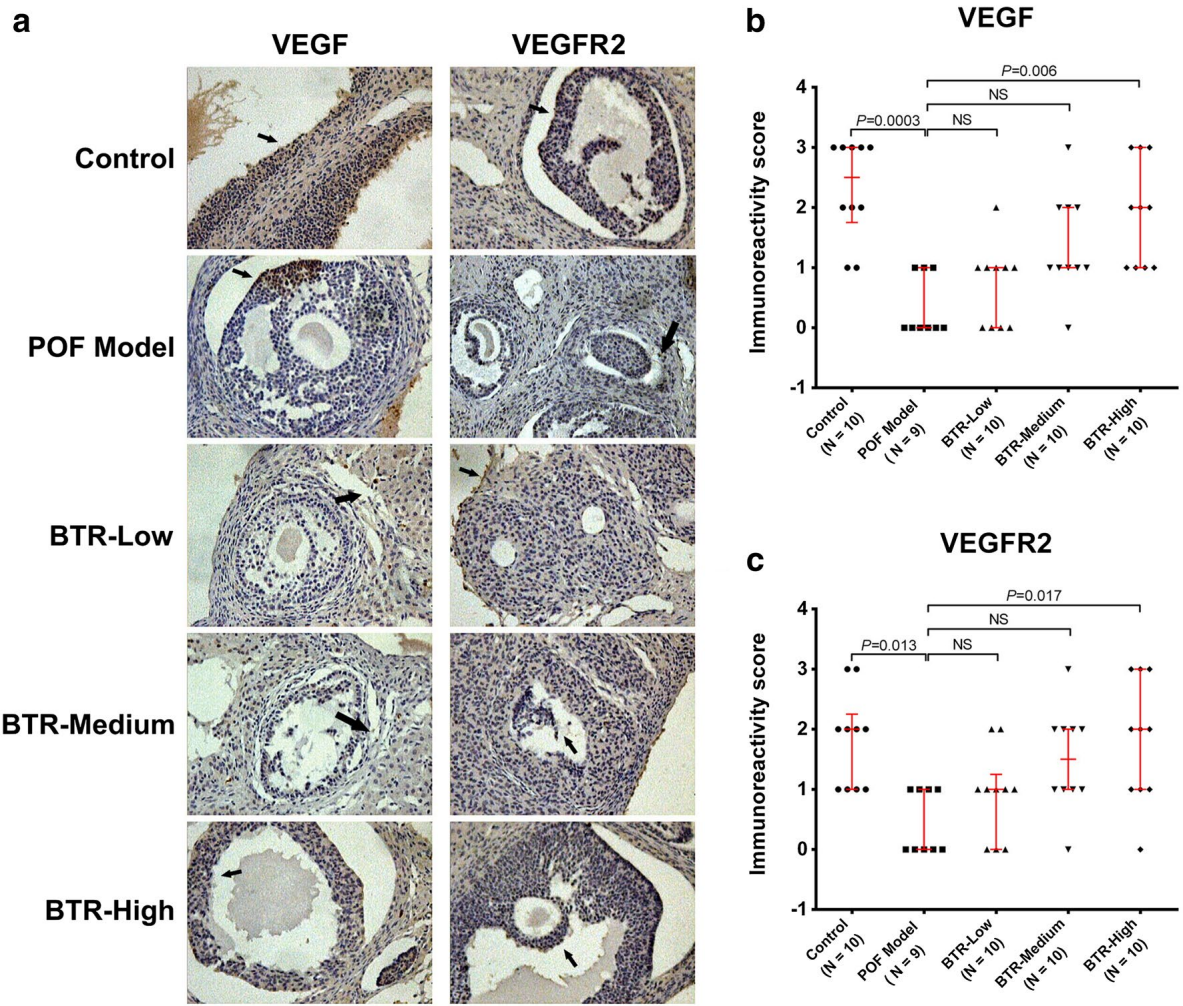
Representative immunohistochemistry images (**a**) and quantitative analysis (**b**, **c**) of VEGF and VEGFR2 in histological sections from all experimental groups. Immunostaining (*brown*) are indicated by *black arrows*. For each animal, ten random high power fields (HPFs) from five sections were used for quantitative analysis. Each *dot* in **b** and **c** represents the median value across these HPFs. *Bars* and *error bars* are medians and quartiles, respectively. All statistical analyses were performed using nonparametric Kruskal–Wallis test followed by Dunn’s post hoc test. *NS* not significant. Magnification ×100

Histologic analyses of the ovarian sections showed morphological changes of vessels in different groups. We therefore performed IHC staining to investigate the effect of BTR on intraovarian expression of two key angiogenic factors, VEGF and VEGFR2, in a TG-induced POF model. As presented in Fig. 3a, samples from the POF model group showed significantly decreased IHC staining intensity, compared to those from the control group; while this decrease was restored by the administration of BTR in a dose-dependent manner. Results of semi-quantitative IHC assessment also confirmed these findings (Fig. 3b, c). Collectively, these data suggest that TG-induced POF is associated with decreased intraovarian expression of VEGF and VEGFR2, whereas treatment with BTR is able to induce intraovarian expression of both proteins, which may contribute to its effects in TG-induced POF model.

### BTR protects granulosa cells from apoptosis in a TG-induced POF rat model

**Fig. 4:**
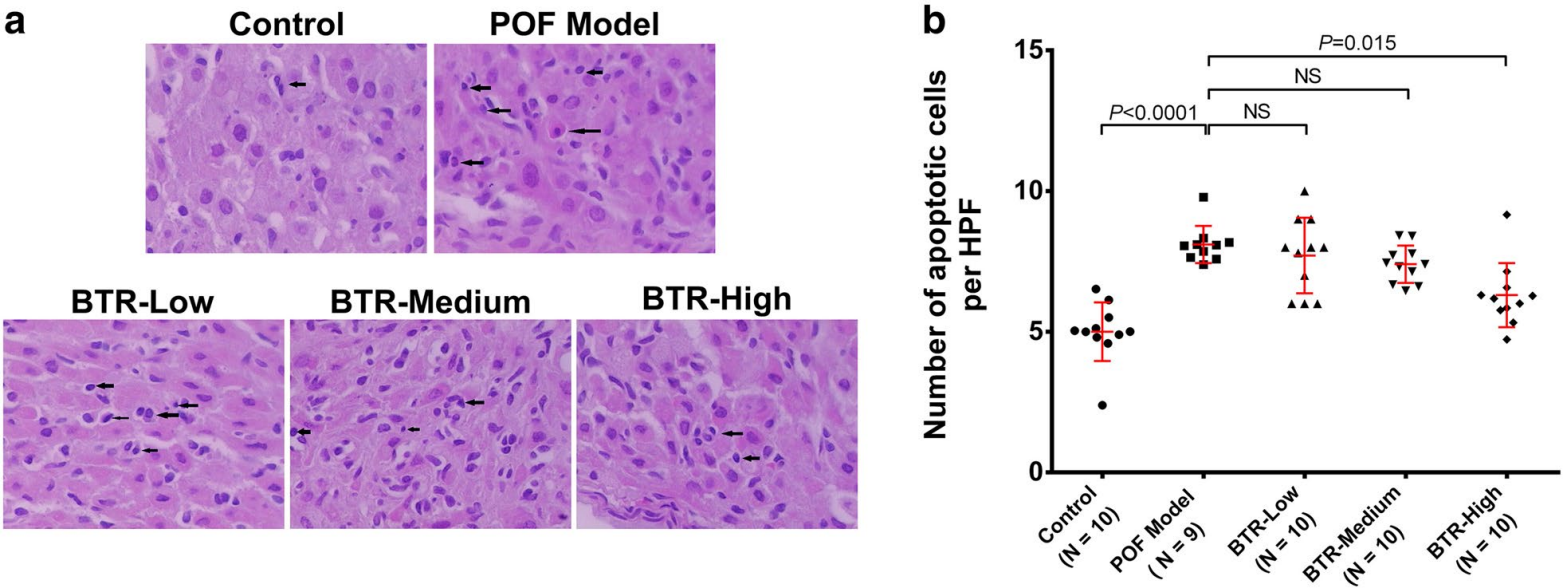
Quantitative assessment of apoptosis in ovarian tissue samples from different experimental groups. **a** Representative hematoxylin and eosin-stained images and **b** apoptotic cell counting results. For each animal, ten high power fields (HPFs) were randomly chosen from five sections for counting the number of apoptotic granulosa cells. Each *dot* in **b** represents the average value across these HPFs. *Bars* and *error bars* are means and SD, respectively. All statistical analyses were performed using ANOVA followed by Tukey’s post hoc test. *NS* not significant. Magnification ×400

Next, we investigated the protective effect of BTR against apoptosis of granulosa cells in a TG-induced POF rat model. H&E stained ovarian sections from the control group showed that most granulosa cells appeared healthy with no sign of apoptosis (Fig. 4a). In the POF model group, however, some cells were compact and irregularly shaped with smaller and condensed nuclei. In addition, the nuclei of cells in advanced stages of apoptosis were fragmented, resulting in vacuolation and apoptotic bodies. Samples from the BTR-treated groups displayed that the majority of granulosa cells had intact cell membranes and clear nuclei (Fig. 4a), and quantitative analysis indicated that the percentage of apoptotic cells was decreased in all BTR-treated groups, especially in the BTR-high group (Fig. 4b). These findings suggest that BTR can protect granulosa cells from apoptosis in a TG-induced POF rat model.

**Fig. 5:**
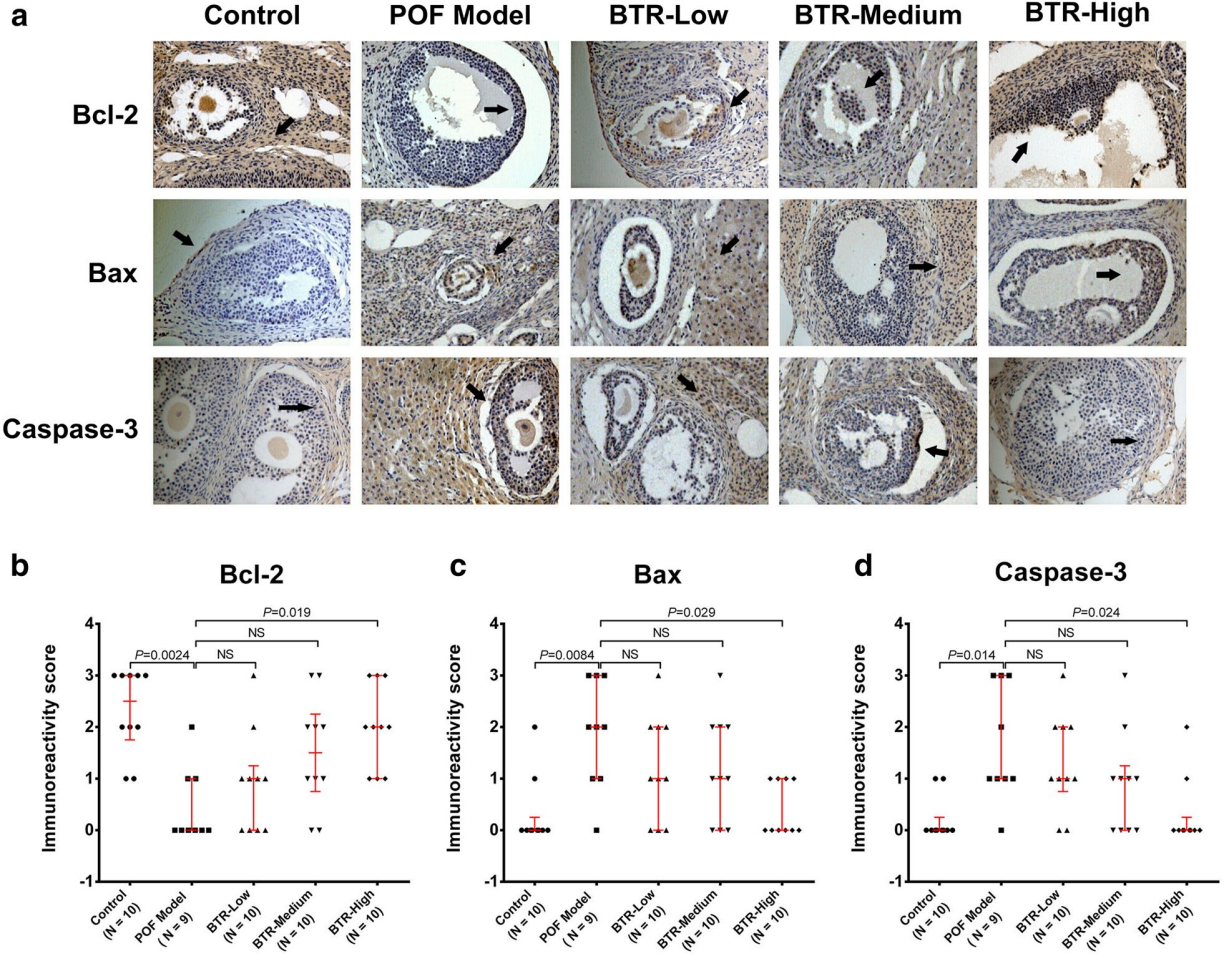
Representative immunohistochemistry images (**a**) and quantitative analysis (**b**, **c**, **d**) of Bcl-2, Bax, and caspase 3 in histological sections from all experimental groups. Immunostaining (*brown*) are indicated by *black arrows*. For each animal, ten random high power fields (HPFs) from five sections were used for quantitative analysis. Each *dot* in **b** and **c** represents the median value across these HPFs. *Bars* and *error bars* are medians and quartiles, respectively. All statistical analyses were performed using the nonparametric Kruskal–Wallis test followed by Dunn’s post hoc test. *NS* not significant. Magnification ×100

In order to further validate the findings of the histological examination, we investigated the effect of BTR on intraovarian expression of several apoptosis-related proteins, Bcl-2, Bax, and caspase-3 in ovarian tissues by IHC staining. As shown in Fig. 5a, samples from the POF model group showed a significantly lower level of Bcl-2 and considerably higher levels of Bax and caspase 3, compared to control ovaries. However, these alterations could be reversed by treatment with BTR in a dose-dependent manner. These findings were also supported by the data of semi-quantitative IHC analysis (Fig. 5b, d), indicating that the effects of BTR treatment may be through regulation of the intraovarian expression of apoptosis-related proteins.

## Discussion

POF is a common cause of infertility in women and is characterized by amenorrhea. It is related to the symptoms and metabolic effects of sex steroid deficiency, as well as the emotional sequelae experienced by couples who have difficulty in conceiving a pregnancy [34]. In addition, POF is associated with risks of cardiovascular disease, osteoporosis, and psychiatric diseases [35–37]. Currently, there are no effective treatments for this disease. Most therapeutic strategies are aimed at relieving the menopausal symptoms, reducing the risk of osteoporosis, and dealing with the loss of fertility [32].

BTR is a TCM formula that has been prescribed by Chinese medicine doctors for decades for the treatment of POF. As compared with other TCM formulas, e.g. Tongmai Dasheng Tablet [38] or Luan-Pao-Prescription [39], BTR only contains four herbs, which may increase safety and reduce treatment cost. In this study, we used a TG-induced POF rat model to investigate the effects of BTR for POF and the underlying mechanisms. TG is an active component of *Tripterygium wilfordii*, which is widely used to treat autoimmune and inflammatory diseases. However, a long-term use of TG can cause irregular menstruation, amenorrhea and even POF [40]. Therefore, the side effects of TG on female reproductive system can be used to induce a POF animal model. Chen et al. [41] have established a mouse POF model via subcutaneous injection of TG, while TG-induced rat POF model has also been established and widely used for reproductive experimental studies [38, 42]. In the current study, we found that BTR not only reverts an abnormal estrous cycle and a decreased ovary index in this model but also improves the abnormal secretion of reproductive hormones associated with POF. In addition, treatment with BTR can protect ovaries from TG-induced damage. Further studies showed that BTR can induce intraovarian expression of VEGF and VEGFR2 and regulate intraovarian expression of apoptosis-related proteins, suggesting that promotion of angiogenesis and anti-apoptosis most likely to contribute to the effects of BTR for POF.

The well-established paradigm of reproduction in mammals holds that the correct formation of the thecal vasculature is necessary to assure a proper nutrition and hormonal supply to the developing follicle [43]. This process involves ovarian angiogenesis and vasculogenesis, which are crucial for folliculogenesis, normal development of the corpus luteum, and maintenance of the function of the mature corpus luteum [44]. When angiogenesis and vasculogenesis are impaired, the ovary becomes hypoxic, which may trigger apoptosis of ovarian cells and follicle atresia, eventually leading to POF [43]. VEGF is considered to be a prime regulator of angiogenesis and vasculogenesis that can induce vascular permeability and promote endothelial cell proliferation, migration, and survival through the interaction with its receptors [45, 46]. The VEGF receptor family consists of three members, VEGFR-1, -2, and -3, of which VEGFR-2 is predominantly involved in mediating the effects of VEGF [47]. Researchers have demonstrated the essential role of VEGF and VEGFR-2 in follicular development, ovulation, and corpus luteum formation [48]. When the intraovarian expression of VEGF and VEGFR-2 is inhibited, follicular development and ovulation may also be suppressed [49]. In this study, TG-induced POF was associated with significantly decreased intraovarian expression of VEGF and VEGFR-2. However, treatment with BTR restored VEGF and VEGFR-2 levels in a dose-dependent manner. This effect may be due to the angiogenic properties of the active components of BTR.

BTR contains four Chinese medical herbs, of which *Rehmanniae Radix Praeparata* is the major active component; it has been prescribed for wound healing and tissue regeneration in Chinese Traditional Medicine for decades. A recent study has revealed that the aqueous extract of *Rehmanniae Radix Praeparata* is effective in promoting diabetic foot ulcer healing in rats through up-regulation of VEGF expression [23]. Additionally, another component of BTR, *Corni Fructus*, contains active substances such as cornel iridoid glycoside, which can also promote angiogenesis by increasing the expression of VEGF and VEGFR-2 [24]. Therefore, the restoration of VEGF and VEGFR-2 expression by BTR may be attributed to, at least in part, the angiogenic effect of these two herbs.

Previous studies have documented that POF is associated with accelerated follicular atresia, which primarily results from apoptosis of granulosa cells [50, 51]. Apoptosis is a physiological process that maintains the homeostasis of adult tissue. In most adult tissues, the occurrence of apoptosis is proportional to the cell proliferation rate. In the ovary, however, a high rate of follicular cell apoptosis without constant stem cell renewal ultimately leads to reproductive senescence [52]. Under pathological conditions, the balance between pro-apoptotic and anti-apoptotic signals is disrupted, and excessive apoptosis results in ovarian disorders characterized by infertility including polycystic ovary syndrome and POF. Usually, apoptosis is a tightly regulated process, which can be initiated via two alternative signaling pathways, the death receptor-mediated “extrinsic apoptotic pathway” and the mitochondrion-mediated “intrinsic apoptotic pathway”. The latter is considered to be involved in the POF process [53]. In the mitochondrion-mediated apoptosis pathway, Bax is a pro-apoptotic protein that promotes the release of cytochrome c (Cyt-c) into the cytosol. Following the release, Cyt-c forms a complex with ATP and apoptotic protease activating factor (Apaf-1), subsequently activating caspase 9, and in turn the executioner caspases, including caspase 3. Recent reports have demonstrated that up-regulation of Bax and caspase 3 can be detected in chemotherapy-induced POF animal models [54, 55]. Additionally, as a key anti-apoptotic factor, Bcl-2 is involved in the regulation of caspase activity and its up-regulation can prevent apoptosis of ovarian granulosa cells [56]. In the current study, we found that treatment with BTR could ameliorate TG-induced apoptotic death of ovarian tissues, which was accompanied by down-regulation of Bax and caspase 3 and up-regulation of Bcl-2. This effect may be attributed to the anti-apoptotic activity of the two components of BTR, *Paeoniae Radix Alba* and *Corni Fructus*. Sun et al. [25] found that the aqueous extract of Radix Paeoniae Alba is able to up-regulate Bcl-2 and down-regulate Bax in PC12 cells. Park et al. [26] reported that phenolic compounds isolated from *Corni Fructus* can ameliorates renal damage by reducing renal expression of pro-apoptotic factors such as Bax and Cyt-c. However, as an aqueous extract of four herbs, BTR is of great complexity in ingredients; the major substances responsible for the anti-apoptotic activity and how they exert their signaling functions requires further investigation.

Several limitations should be addressed regarding the current study. First, although TG-induced POF rat model is widely used, it does not fully reflect the pathogenetic process of POF. Therefore, the findings of this study still need to be validated in other POF animal models (e.g. natural aging POF rat model). Second, the results of mechanistic study are still preliminary; additional studies are necessary to elaborate the precise signaling pathways that are involved in the pro-angiogenic and anti-apoptotic effects of BTR. Additionally, it should be noted that there was a great discrepancy with previous literature as to the serum level of E2 in experimental animals [38, 39], which may be due to differences in detection methods or assay kits across the studies. This indicates that the assay of serum E2 level should be carefully optimized in the future.

## Conclusion

In summary, the current study shows that BTR is effective for the treatment of TG-induced POF rats. Results of histological and IHC analyses suggest promotion of angiogenesis and anti-apoptosis are the two possible mechanisms accounting for the effects of BTR. These findings provide new insights into the molecular mechanisms whereby BTR improves POF.

## Additional files

**Additional file 1.** Minimum standards checklist for confirming the information of methods.

**Additional file 2.** Additional data.

## Abbreviations

ANOVA: one-way analysis of variance
BTR: Bushen Tianjing Recipe
DAB: 3,3′-diaminobenzidine
E2: estradiol
FSH: follicle-stimulating hormone
GnRH: gonadotropin-releasing hormone
H&E: hematoxylin and eosin
HPFs: high power fields
HRT: hormone replacement therapy
IHC: immunohistochemistry
IQR: interquartile range
P: progesterone
POF: premature ovarian failure
SD: standard deviation
T: testosterone
TCM: Traditional Chinese Medicine
VEGF: vascular endothelial growth factor
VEGFR2: vascular endothelial growth factor receptor 2

## References

[1] Coulam CB, Adamson SC, Annegers JF (1986). Incidence of premature ovarian failure. Obstet Gynecol.

[2] Kokcu A (2010). Premature ovarian failure from current perspective. Gynecol Endocrinol.

[3] Nelson LM (2009). Clinical practice. Primary ovarian insufficiency. N Engl J Med.

[4] Kalantaridou SN, Davis SR, Nelson LM (1998). Premature ovarian failure. Endocrinol Metab Clin N Am.

[5] Woad KJ, Watkins WJ, Prendergast D, Shelling AN (2006). The genetic basis of premature ovarian failure. Aust N Z J Obstet Gynaecol.

[6] Goswami D, Conway GS (2005). Premature ovarian failure. Hum Reprod Update.

[7] Rebar RW (2009). Premature ovarian failure. Obstet Gynecol.

[8] Alzubaidi NH, Chapin HL, Vanderhoof VH, Calis KA, Nelson LM (2002). Meeting the needs of young women with secondary amenorrhea and spontaneous premature ovarian failure. Obstet Gynecol.

[9] Welt CK (2008). Primary ovarian insufficiency: a more accurate term for premature ovarian failure. Clin Endocrinol.

[10] Roberts H (2007). Managing the menopause. BMJ.

[11] Wang C, Chen M, Fu F, Huang M (2013). Gonadotropin-releasing hormone analog cotreatment for the preservation of ovarian function during gonadotoxic chemotherapy for breast cancer: a meta-analysis. PLoS ONE.

[12] Badawy A, Elnashar A, El-Ashry M, Shahat M (2009). Gonadotropin-releasing hormone agonists for prevention of chemotherapy-induced ovarian damage: prospective randomized study. Fertil Steril.

[13] Bidet M, Bachelot A, Touraine P (2008). Premature ovarian failure: predictability of intermittent ovarian function and response to ovulation induction agents. Curr Opin Obstet Gynecol.

[14] Beal MW (1998). Women’s use of complementary and alternative therapies in reproductive health care. J Nurse Midwifery.

[15] Huang ST, Chen AP (2008). Traditional Chinese medicine and infertility. Curr Opin Obstet Gynecol.

[16] Sze SC, Cheung HP, Ng TB, Zhang ZJ, Wong KL, Wong HK, Hu YM, Yow CM, Tong Y (2011). Effects of Erxian decoction, a Chinese medicinal formulation, on serum lipid profile in a rat model of menopause. Chin Med.

[17] Mohammad-Alizadeh-Charandabi S, Shahnazi M, Nahaee J, Bayatipayan S (2013). Efficacy of black cohosh (*Cimicifuga racemosa* L.) in treating early symptoms of menopause: a randomized clinical trial. Chin Med.

[18] Wang S, Tong Y, Ng TB, Lao L, Lam JK, Zhang KY, Zhang ZJ, Sze SC (2015). Network pharmacological identification of active compounds and potential actions of erxian decoction in alleviating menopause-related symptoms. Chin Med.

[19] Liang Y, Du HL, Chang XF, Zhao SN, Lei LM (2014). Effect of Bushen Tiaojing Recipe on the quality of the oocytes and reproductive hormones in the follicular fluid in IVF-ET patients. Zhongguo Zhong Xi Yi Jie He Za Zhi.

[20] Xu BH, Li MQ, Luo YJ (2013). Treatment of premature ovarian failure patients by Bushen Tiaojing Recipe combined hormone replacement therapy: a clinical observation. Zhongguo Zhong Xi Yi Jie He Za Zhi.

[21] Wei M, Lu Y, Liu D, Ru W (2014). Ovarian failure-resistant effects of catalpol in aged female rats. Biol Pharm Bull.

[22] Takeuchi T, Nishii O, Okamura T, Yaginuma T (1991). Effect of paeoniflorin, glycyrrhizin and glycyrrhetic acid on ovarian androgen production. Am J Chin Med.

[23] Lau TW, Lam FF, Lau KM, Chan YW, Lee KM, Sahota DS, Ho YY, Fung KP, Leung PC, Lau CB (2009). Pharmacological investigation on the wound healing effects of Radix Rehmanniae in an animal model of diabetic foot ulcer. J Ethnopharmacol.

[24] Yao RQ, Zhang L, Wang W, Li L (2009). Cornel iridoid glycoside promotes neurogenesis and angiogenesis and improves neurological function after focal cerebral ischemia in rats. Brain Res Bull.

[25] Sun R, Wang K, Wu D, Li X, Ou Y (2012). Protective effect of paeoniflorin against glutamate-induced neurotoxicity in PC12 cells via Bcl-2/Bax signal pathway. Folia Neuropathol.

[26] Park CH, Noh JS, Tanaka T, Yokozawa T (2012). 7-*O*-Galloyl-d-sedoheptulose ameliorates renal damage triggered by reactive oxygen species-sensitive pathway of inflammation and apoptosis. J Pharm Pharmacol.

[27] Tropp J, Markus EJ (2001). Effects of mild food deprivation on the estrous cycle of rats. Physiol Behav.

[28] Reagan-Shaw S, Nihal M, Ahmad N (2008). Dose translation from animal to human studies revisited. FASEB J.

[29] Westwood FR (2008). The female rat reproductive cycle: a practical histological guide to staging. Toxicol Pathol.

[30] Fraser EJ, Shah NM (2014). Complex chemosensory control of female reproductive behaviors. PLoS ONE.

[31] Bhavina K, Radhika J, Pandian SS (2014). VEGF and eNOS expression in umbilical cord from pregnancy complicated by hypertensive disorder with different severity. Biomed Res Int.

[32] Shelling AN (2010). Premature ovarian failure. Reproduction.

[33] Ebrahimi M, Asbagh FA (2011). Pathogenesis and causes of premature ovarian failure: an update. Int J Fertil Steril.

[34] Kovanci E, Schutt AK (2015). Premature ovarian failure: clinical presentation and treatment. Obstet Gynecol Clin N Am.

[35] Bruning PF, Pit MJ, de Jong-Bakker M, van den Ende A, Hart A, van Enk A (1990). Bone mineral density after adjuvant chemotherapy for premenopausal breast cancer. Br J Cancer.

[36] Carter J, Rowland K, Chi D, Brown C, Abu-Rustum N, Castiel M, Barakat R (2005). Gynecologic cancer treatment and the impact of cancer-related infertility. Gynecol Oncol.

[37] Jeanes H, Newby D, Gray GA (2007). Cardiovascular risk in women: the impact of hormone replacement therapy and prospects for new therapeutic approaches. Expert Opin Pharmacother.

[38] Fu Y, Zhao Z, Wu Y, Wu K, Xu X, Liu Y, Tong C (2012). Therapeutic mechanisms of Tongmai Dasheng Tablet on tripterygium glycosides induced rat model for premature ovarian failure. J Ethnopharmacol.

[39] Jiang B, Sun K, Li M, Wang Y, Zhuang L, Zhang L, Wang Y, Liu X, Wu W, Guan S (2012). Study of Luan-Pao-Prescription on ovarian dysfunction in rats. J Ethnopharmacol.

[40] Chen X, Chen SL (2011). A woman with premature ovarian failure induced by *Tripterygium wilfordii* Hook. f. gives birth to a healthy child. Fertil Steril.

[41] Chen XY, Gu C, Ma M, Cong Q, Guo T, Ma D, Li B (2014). A mouse model of premature ovarian insufficiency induced by tripterygium glycoside via subcutaneous injection. Int J Clin Exp Pathol.

[42] Su J, Ding L, Cheng J, Yang J, Li X, Yan G, Sun H, Dai J, Hu Y (2016). Transplantation of adipose-derived stem cells combined with collagen scaffolds restores ovarian function in a rat model of premature ovarian insufficiency. Hum Reprod.

[43] Malamitsi-Puchner A, Sarandakou A, Tziotis J, Stavreus-Evers A, Tzonou A, Landgren BM (2004). Circulating angiogenic factors during periovulation and the luteal phase of normal menstrual cycles. Fertil Steril.

[44] Stouffer RL, Xu F, Duffy DM (2007). Molecular control of ovulation and luteinization in the primate follicle. Front Biosci.

[45] Araujo VR, Duarte AB, Bruno JB, Pinho Lopes CA, de Figueiredo JR (2013). Importance of vascular endothelial growth factor (VEGF) in ovarian physiology of mammals. Zygote.

[46] Dvorak HF, Nagy JA, Feng D, Brown LF, Dvorak AM (1999). Vascular permeability factor/vascular endothelial growth factor and the significance of microvascular hyperpermeability in angiogenesis. Curr Top Microbiol Immunol.

[47] Lam PM, Haines C (2005). Vascular endothelial growth factor plays more than an angiogenic role in the female reproductive system. Fertil Steril.

[48] Robinson RS, Woad KJ, Hammond AJ, Laird M, Hunter MG, Mann GE (2009). Angiogenesis and vascular function in the ovary. Reproduction.

[49] Fraser HM (2006). Regulation of the ovarian follicular vasculature. Reprod Biol Endocrinol.

[50] Fotovati A, Abu-Ali S, Nakayama K, Nakayama KI (2011). Impaired ovarian development and reduced fertility in female mice deficient in Skp2. J Anat.

[51] Zhao XJ, Huang YH, Yu YC, Xin XY (2010). GnRH antagonist cetrorelix inhibits mitochondria-dependent apoptosis triggered by chemotherapy in granulosa cells of rats. Gynecol Oncol.

[52] Hsueh AJ, Billig H, Tsafriri A (1994). Ovarian follicle atresia: a hormonally controlled apoptotic process. Endocr Rev.

[53] Kappeler CJ, Hoyer PB (2012). 4-Vinylcyclohexene diepoxide: a model chemical for ovotoxicity. Syst Biol Reprod Med.

[54] Vujovic S (2009). Aetiology of premature ovarian failure. Menopause Int.

[55] Xia T, Fu Y, Gao H, Zhao Z, Zhao L, Han B (2014). Recovery of ovary function impaired by chemotherapy using Chinese herbal medicine in a rat model. Syst Biol Reprod Med.

[56] Kaipia A, Hsu SY, Hsueh AJ (1997). Expression and function of a proapoptotic Bcl-2 family member Bcl-XL/Bcl-2-associated death promoter (BAD) in rat ovary. Endocrinology.

