# Supplementary material for: Effects of Bushen Tianjing Recipe in a rat model of tripterygium glycoside-induced premature ovarian failure
Source: Chin Med. 2017 Apr 24;12:10. doi: 10.1186/s13020-017-0131-3 (PMC5402324; doi:10.1186/s13020-017-0131-3)

*Supplementary Information*

Effects of Bushen Tianjing Recipe in a rat model of tripterygium glycoside-induced premature ovarian failure

Xiaofeng Xu^1^, Yong Tan^2^, Guorong Jiang^1^, Xuanyi Chen^1,^*, Rensheng Lai^3^, Lurong Zhang^1^, Guoqiang Liang^1^

**^1^** Department of Obstetrics and Gynecology, Suzhou Hospital Affiliated to Nanjing University of Chinese Medicine, Suzhou, Jiangsu Province 215009, China

**^2^** Department of Gynecology, the No.1 Clinical Medical College, Nanjing University of Chinese Medicine, Nanjing, Jiangsu Province 210029, China

*^3^ Department of Pathology, Suzhou Hospital Affiliated to Nanjing University of Chinese Medicine, Suzhou, Jiangsu Province 215009, China*

* Corresponding Author: Xuanyi Chen, Professor of Medicine

Affiliation: Department of Obstetrics and Gynecology, Suzhou Hospital Affiliated to Nanjing University of Chinese Medicine, Suzhou, Jiangsu Province 215009, China

Address: No. 18 Yangsu Road, Gusu District, Suzhou, Jiangsu Province 215009, China

Tel: +86-13584890560

Fax: N/A

Email: chenxuanyi1212@sohu.com

Materials and Methods

Chemicals and reagents

The reference standards of catalpol, paeoniflorin, morroniside, and arginine were purchased from the National Institutes for Food and Drug Control (Beijing, China). The high performance liquid chromatogram (HPLC) column was from Thermo Fisher Scientific (Hypersil^TM^ C18 Column, Waltham, MA, USA). The ultraviolet detector was from Hitachi (U-3300, Tokyo, Japan). All other solvents (HPLC grade) were purchased from Sigma-Aldrich, China (Shanghai, China).

Procedures of high performance liquid chromatogram

The procedures of high performance liquid chromatogram (HPLC) were as follows. First, 1 g BTR was dissolved in 80 % methanol (v/v) with the aid of ultrasonication for 30 min. Then, the resulting solution was filtered using a membrane filer (pore size = 0.25 μm, Merck Millipore, ‎Billerica, MA, USA), and 10 μl filtered solution was injected into the HPLC system (Agilent 1100 HPLC System, Santa Clara, CA, USA) for analysis. The reference standards of catalpol, paeoniflorin, and morroniside were also dissolved in 80% methanol at concentration of 50 μg/ml. The HPLC conditions for catalpol were: the mobile phase, 0.1% phosphate buffer; the flow rate, 1.0 ml/min; the wavelength of detection, 210 nm; and the column temperature, 25 ºC. The HPLC conditions for paeoniflorin were: the mobile phase, methanol-water-phosphate buffer (28: 72: 0.1, v/v); the flow rate, 1.0 ml/min; the wavelength of detection, 230 nm; and the column temperature, 25 ºC. The HPLC conditions for morroniside were: the mobile phase, methanol-water-phosphate buffer (28: 72: 0.1, v/v); the flow rate, 1.0 ml/min; the wavelength of detection, 250 nm; and the column temperature, 25 ºC. The contents of catalpol, paeoniflorin, and morroniside were calculated from the area under the peak, at the base of their individual external standards.

Procedures of thin layer chromatography

Thin layer chromatography (TLC) was used to detect the presence of arginine, an active component of *Testudinis Carapax et Plastrum*, in BTR according to the method described in previous literature [1]. Briefly, standard arginine solution (1 mg/ml) was prepared in 0.01 M phosphate buffer (pH 8.0). Then, 1 µl BTR and 1 µl standard arginine solution were spotted on the TLC plates (Silica gel 60G F₂₅₄, Merck Millipore, Catalog No. 1003900001). Plates were air-dried and then developed using n-propanol-water (70: 30, v/v) as the mobile phase. After development, plates were dried and visualized with 0.25% ninhydrin in acetone.

References

1. Sen S, Sarkar S, Kundu P, Laskar S. Separation of amino acids based on thin-layer chromatography by a novel quinazoline based anti-microbial agent. Am J Analyt Chem. 2012; 3: 669-74.

Figure Legends

**Supplementary Figure 1**. The representative HPLC chromatogram of catalpol in BTR. A: The reference standard of catalpol; B: BTR sample (batch No.: BSTJ001).

**Supplementary Figure 2**. The representative HPLC chromatogram of paeoniflorin in BTR. A: The reference standard of paeoniflorin; B: BTR sample (batch No.: BSTJ001).

**Supplementary Figure 3**. The representative HPLC chromatogram of morroniside in BTR. A: The reference standard of morroniside; B: BTR sample (batch No.: BSTJ001).

**Supplementary Figure 4**. The representative TLC chromatogram of arginine in BTR. Lane A: the reference standard of arginine; lane B: 0.01 M phosphate buffer (pH 8.0) as negative control; lane C: BTR sample (batch No.: BSTJ001).

**Supplementary Figure 5**. Quantitative analysis of numbers of follicles in BTR treated animals. For each animal, 10 high power fields (HPFs) were randomly chosen from five sections for counting the number of follicles (both primordial and primary). Each dot represents the average value across these HPFs. Bars and error bars are means and SD, respectively. All statistical analyses were performed using ANOVA followed by Tukey’s post-hoc test. NS indicates not significant.

**Supplementary Table 1. HPLC and TLC analyses of three batches of BTR**

| **Batch No.** | **BSTJ001** | **BSTJ001** | **BSTJ001** | **Average** | **RSD (%)** |
| --- | --- | --- | --- | --- | --- |
| Content of catalpol  (mg/10 g BTR) | 55 | 59 | 56 | 57 | 3.7 |
| Content of paeoniflorin  (mg/10 g BTR) | 220 | 237 | 241 | 233 | 4.8 |
| Content of morroniside  (mg/10 g BTR) | 62 | 61 | 65 | 63 | 3.3 |
| TLC analysis of arginine | Positive | Positive | Positive | N/A | N/A |

BTR, Bushen Tianjing Recipe; RSD, relative standard deviation

**Figure 1**





**Figure 2**





**Figure 3**


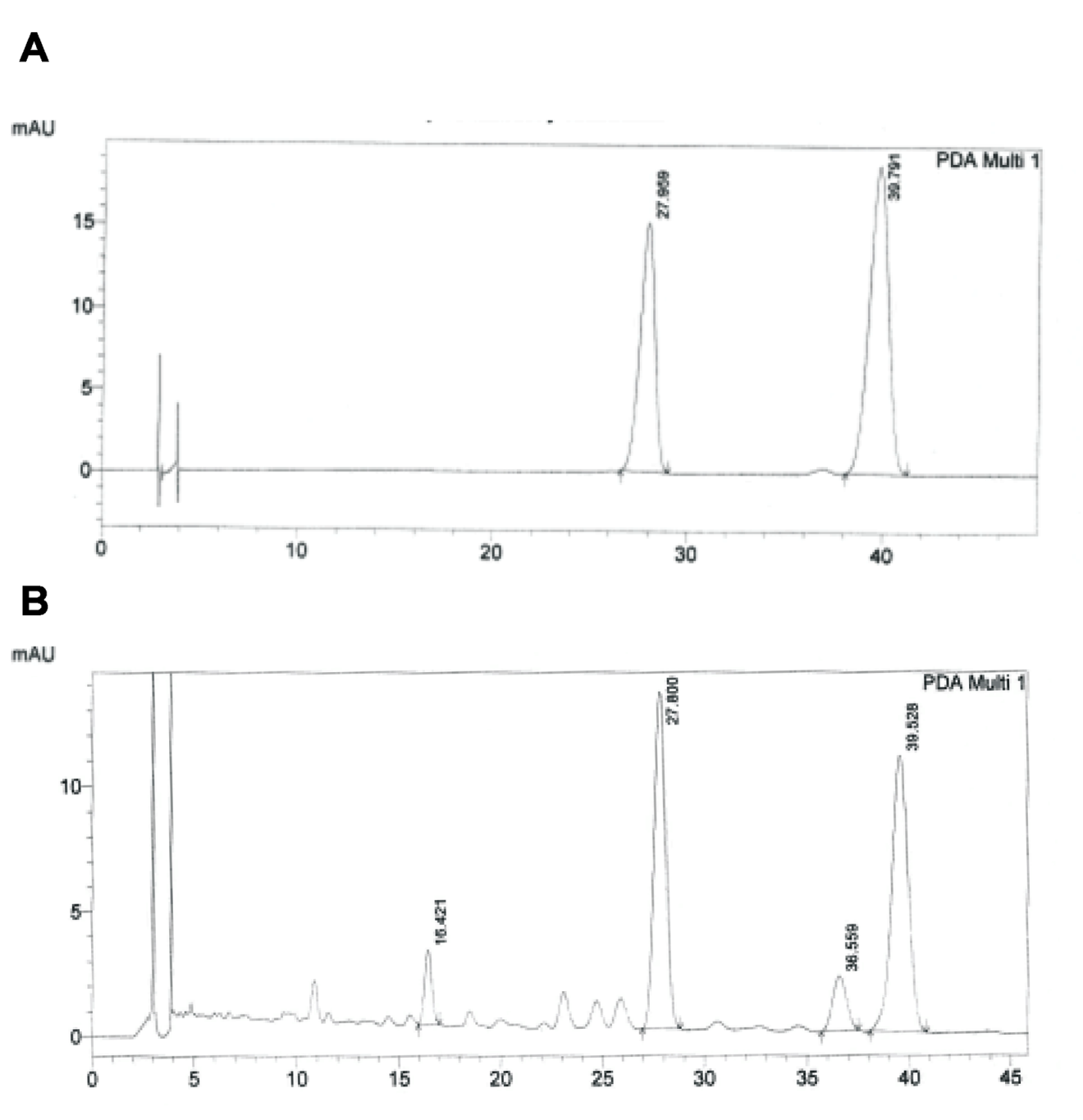


**Figure 4**


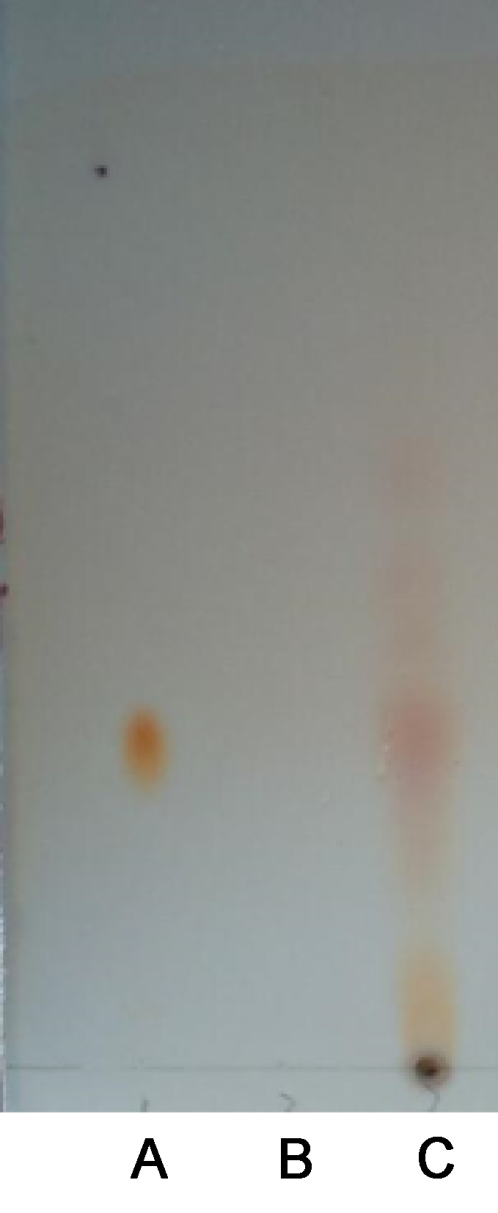


**Figure 5**


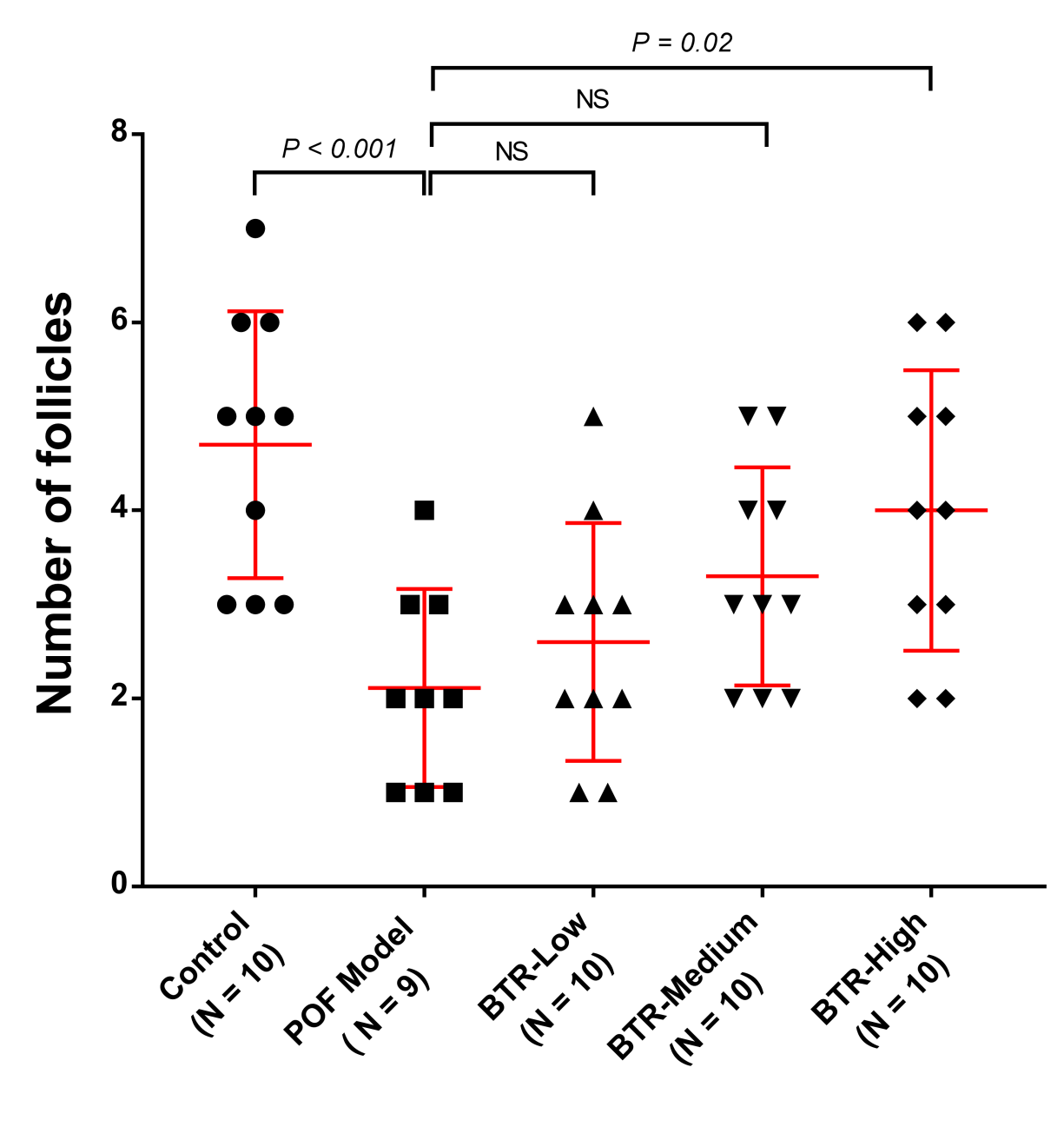

Supplement: Supplementary file 2 — Additional file 2. Additional data. [file 13020_2017_131_MOESM2_ESM.docx]
